# Nerve-Tides

**Published:** 1888-04-14

**Authors:** 


### The Doctor's Pulpit.

### NERVE-TIDES.

Last week nerve-storms were talked about; this week it may be worth while to devote a little attention to nerve-tides. In the order of logic the tides should properly have come first, but as nerve-storms have been for some time before the public, whilst nerve-tides belong to a more recent period of nomenclature, the former have established a kind of vested right to precedence. "There is a tide in the affairs" of nerves as of men, which it is well to take at the flood, and which so taken, habitually, "leads on to"—health. The nervous energy has its regular ebb and flow. The full tide is the time to set sail for work, whether mental or physical; the ebb tide is the time to cast anchor. Let it never be forgotten that the human organism is not like a steamship, which, so long as it is well coaled, can sail by night as well as by day. Rather may it be compared with the little fishing-boat, which has its daily periods of industry within a few miles of the shore, and its similar periods of lying high and dry on the beach.

In a healthy man the nerve-tide is at its height in the morning, probably soon after breakfast. There is at that period a flow of spirits, a hopeful animation, a sense of capacity and courage, a feeling of "swing" and of "go," which is as delightful as it is full of working power. If possible a man should do his best work then; he should so arrange his business as to let his most important duties fall at that time. This remark applies particularly to intellectual workers—clergymen, authors, barristers, and reading doctors. The "midnight oil" used to be an approved phrase, and the parson whose sermons smelt of it was believed to be a deeper thinker and a finer preacher than his neighbours. To our thinking the sermons of such preachers do not possess anything like the illuminating power of discourses produced in the early morning light, but rather reflect the sickly glare of a badly-cleaned paraffin lamp.

It may happen that, from habit, a man not only works best at midnight, but even that he cannot work well with his brain at any other time. Possibly in some cases attempts to break off such habits would prove complete failures. Such exceptions notwithstanding, the habit is a bad one. We do not advise the clergyman of fifty, who has practised it for a quarter of a century, to compel himself to change; but all men short of that age, and emphatically all young men, should learn to work in the morning. Then is the time for easy thinking, then is the time for clearness, then the time for strength, and then the time for the exercise of that finest and most wholesome of all literary or theological qualities—a "saving common sense."

After mid-day the tide of nerve energy will begin to turn. Then a meal should be taken. This particular meal is a very important consideration for a man of working habits. If it be too heavy, the eater will be becalmed, for he will go to sleep, or want to do so. If it be insufficient, it will make him irritable and ill-tempered; his barque will sail on a "choppy" sea all the afternoon. The kind of meal that is wanted is one which shall be "light" but "satisfying." What such a meal is to consist of each one must find out for himself.

Afternoon is the time for routine work—work which demands no special intellectual freshness or force. Ladies may then make their morning calls, business letters of third-rate importance may then be written. The young woman who is tired of her lover should make a point of seeing him only in the afternoon. He will, probably, in that case soon be equally tired of her, and the engagement may be gently broken off with mutual satisfaction. There is an ebbing of the nerve-tide in the afternoon, and it is unprofitable to set all sail. Towards evening the tide becomes quite low, and then is the proper time to leave off the work of the day.

In spite of what our Elizabethan forefathers said and did to the contrary, and notwithstanding the opinions of some eminent physicians of recent times, the present writer strongly holds the view that evening is the rational time to dine. There should only be two really substantial meals a-day, and those should be breakfast and dinner. A solid and highly-nutritious meal ought to begin the day's work; an equally solid and equally nutritious meal should end it. What is taken in the course of the working hours may be such as merely to satisfy the urgent cravings of the appetite, and to maintain in a condition of steady movement the ascending or descending course of the nerve energy.

After dinner—rest; but rational, intelligent rest. Not the rest of the pig in the sty, who gorges until he can gorge no more, and then sleeps as if he would wake no more. The dinner should be a good meal—a nutritious and satisfying meal. But it should not be heavy and sleep-producing. In the early evening the nerve-tide is at its lowest ebb; but later on, and long before bedtime, it has begun to flow again, and keeps on rising—other things being equal—until the time for sleep, or later. It is as if Nature intended us not only to have a flowing tide for work, but a flowing tide for pleasure also. The evening ought to be devoted to rest and animated recreation. Young people have the sense to use it for these purposes, therein showing themselves much wiser than their elders. This is the order of Nature; this is the order to which, if a man conform himself, he will in a lifetime do the most and the best work of which his particular mental and physical organism is capable.

Some sailors are not wise. Instead of using the tides as they find them, with reasonable submission to the inevitable, they often fight against them unnecessarily, and not seldom suffer for their pains. It is even so and worse with those who ought to work with the nerve-tides, and never, except on the sternest compulsion, against them. There are men who, no sooner do they find the tide rising again after dinner, than they immediately rush back to work, and keep at it without intermission until the small hours of the morning. Mad sailors these! If their barque does not founder in mid-ocean it will be through no skill of theirs! No man is stronger than the laws of Nature—no man is so strong. Neither a Wellington, nor a Napoleon, nor a Bismarck, nor a Lesseps, can fight successfully against the established order. The sea laughed at Canute as he laughed at his courtiers, and so will all the tides of fixed order and natural law defy and finally destroy the foolish men and women who vainly think to resist them.

